# miRNAs: biological and clinical determinants in epilepsy

**DOI:** 10.3389/fnmol.2015.00059

**Published:** 2015-10-13

**Authors:** Walid A. Alsharafi, Bo Xiao, Mutasem M. Abuhamed, Zhaohui Luo

**Affiliations:** ^1^Department of Neurology, Xiangya Hospital, Central South UniversityChangsha, China; ^2^Department of Neurology, Alkhadamat Altebia HospitalAmman, Jordan

**Keywords:** epilepsy, miRNA, pathogenesis, hippocampus, biomarkers

## Abstract

Recently, microRNAs (miRNAs) are reported to be crucial modulators in the pathogenesis and potential treatment of epilepsies. To date, several miRNAs have been demonstrated to be significantly expressed in the epileptic tissues and strongly associated with the development of epilepsy. Specifically, miRNAs regulate synaptic strength, inflammation, neuronal and glial function, ion channels, and apoptosis. Furthermore, peripheral blood miRNAs can also be utilized as diagnostic biomarkers to assess disease risk and treatment responses. Here, we will summarize the recent available literature regarding the role of miRNAs in the pathogenesis and treatment of epilepsy. Moreover, we will provide brief insight into the potential of miRNA as diagnostic biomarkers for early diagnosis and prognosis of epilepsy.

## Introduction

Epilepsy is a prevalent chronic neurologic disorder characterized by recurrent unprovoked seizures due to abnormal neuronal excitability (Hauser and Kurland, 1975). However, there is growing concern that the pathogenic epilepsy mechanism remains poorly defined. It is estimated that 50 million patients have a diagnosis of epilepsy worldwide with almost 400,000 new cases of epilepsy diagnosed each year. Unfortunately, one-third of patients are resistant to currently available antiepileptic drugs (AEDs) that act primarily on the brain to reduce the frequency and severity of seizures (Perucca et al., 2007). Over the past five decades, there has been a scarcity of novel treatments that significantly avoid or reverse the development of epilepsy. Therefore, efforts are currently underway to identify alternative therapeutic approaches that can be utilized either individually or in combination with other AEDs to drastically improve the morbidity and mortality associated with epilepsy. Many recent studies have provided evidence for the potential role of miRNAs in the pathogenesis and treatments of epilepsy. Most of these studies employed animal models of epilepsy (Liu et al., 2010; Hu et al., 2011, 2012a; Jimenez-Mateos et al., 2011; Song et al., 2011; McKiernan et al., 2012; Pichardo-Casas et al., 2012; Bot et al., 2013; Risbud and Porter, 2013; Gorter et al., 2014; Li et al., 2014; Kretschmann et al., 2015), whereas relatively few studies used human epileptic samples (Kan et al., 2012; McKiernan et al., 2012b; Kaalund et al., 2014; Zucchini et al., 2014; Wang et al., 2015a,b).

Multiple animal models have been extensively employed to study the pathogenesis and treatment of epilepsy. These epileptic models can be induced by chemoconvulsants, electrical stimulation, brain pathology, or genetic engineering. For an in-depth review of animal models of epilepsy the reader is referred to recent excellent reviews (Löscher, 2011; Kandratavicius et al., 2014). Experimental models induced by chemoconvulsants, primarily kainic acid (KA) and pilocarpine, are the most widespread popular and accepted models in the scientific community, not only because they are convenient, but also because they are easy to implement (Leite et al., 2002). These models are able to evoke both acute and chronic episodes of status epilepticus (SE) with a latent phase between them. It is worth to not that experimental model induced either by KA or pilocarpine is considered more appropriate for TLE studies, due to their seizure-related neuronal injury and hippocapmal synaptic reorganization (Lothman and Bertram, 1993; Mathern et al., 2002). Kindled animals, an electrical stimulated model, are appropriate for investigating seizure thresholds and therapeutic responses. It resembles complex partial seizures with secondary generalization. However, kindling and its relevance to human epilepsy are still debatable (Bertram, 2007).

Historically, different species have been used for epilepsy studies such as mice, rats, rabbits, guinea pigs, cats, and dogs. Compared with other species, rodents (mice and rats) are the most widely employed species in the field of epilepsy. Specific similarities and differences results have shown between humans and each of these species.

MiRNAs are a group of endogenous noncoding ribonucleic acids (RNA) that mainly function as key modulators of gene expression at the posttranscriptional level (Pasquinelli, 2012; Ha and Kim, 2014). Mature miRNAs are able to inhibit messenger RNA (mRNA) translation and/or promoting mRNA degradation via binding usually to complementary sequences within the 3′ untranslated region (3′ UTR) (Lee et al., 1993; Wightman et al., 1993). However, miRNAs have also been reported to bind to other positions. Notably, single miRNA can target several hundred genes and an individual gene can be targeted by multiple miRNAs. In the last two decades, miRNAs have fundamentally changed scientists' understanding of gene regulatory network (GRN) representing a new interdisciplinary direction in neuroscience research. To date, several thousands of miRNAs have been identified as key modulators of various GRN. Recently, numerous studies have shown that miRNAs regulate a wide spectrum of brain functions not only in brain development but also in pathogenic conditions such as epilepsy (Barca-Mayo and De Pietri Tonelli, 2014). Multiple lines of evidence indicate that miRNAs participate in the pathogenesis, treatment, and prevention of epilepsy. They can negatively modulate an enormous and complex regulatory network of gene expression levels that govern the process of epilepsy either in experimental model (Liu et al., 2010; Hu et al., 2011, 2012a; Jimenez-Mateos et al., 2011; Song et al., 2011; McKiernan et al., 2012; Pichardo-Casas et al., 2012; Bot et al., 2013; Risbud and Porter, 2013; Gorter et al., 2014; Li et al., 2014; Kretschmann et al., 2015) or human epilepsy (Kan et al., 2012; McKiernan et al., 2012b; Kaalund et al., 2014; Zucchini et al., 2014; Wang et al., 2015a,b). Therefore, deeper comprehension of the roles, targets, and mechanisms of miRNAs in the pathogenesis and pathophysiology of epilepsy will both develop diagnostic biomarkers for epilepsy and identify promising therapeutic strategies for the development of novel treatments for epilepsy.

Extensive efforts to get better understanding of miRNA biology have yielded several publications and databases of functional miRNA information. In this review, we will focus on the role of miRNAs regarding their function in the pathogenesis of epilepsy and summarize the potential utility of miRNA biology in epilepsy.

## Biology of miRNAs

MiRNAs comprise species of small endogenous non-coding RNAs molecules which generally target one or more mRNAs to control gene expression either by inducing mRNA decay or reducing its translation. However, miRNAs may positively modulate mRNA translation through target gene promoter binding.

It is well-established that mRNA translation is locally modulated by miRNAs within the synaptodendritic compartment in response to RICS activity (Im and Kenny, 2012; Aksoy-Aksel et al., 2014). This indicates the spatial- specificity of expression of the corresponding genes. Of these, some expressed in particular subcellular compartments, cell types, and/or neuroanatomical areas playing critical roles in the brain development and homeostasis (Cao et al., 2006; Cohen et al., 2011). Alteration of miRNA expression during SE shows that modulation of miRNAs levels may participate in the alterations in protein expression during disease progression. Recent study reported that SE may inhibit synaptoneurosome miRNAs, which affect the reaction of synapses (Risbud and Porter, 2013). Moreover, dynamic regulation in the local miRNAs from brain may be involved in the regulation of synaptic protein synthesis (Pichardo-Casas et al., 2012).

Exosomes are small secreted lipoprotein vesicles present in most circulating body fluids, including cerebrospinal fluid (CSF) and serum (Keller et al., 2006). They are released from various cells such as dendritic cells and neurons to contribute to cell-to-cell communication and protein and RNA delivery. In CNS, exsosomal protein could be used as biomarkers because it can influence neurons to regulate synaptic scaling and trans-synaptic propagation of pathogenic processes (Saman et al., 2012). It is well established that exosomes contain numerous molecular components of their cell of origin, including mRNAs and miRNAs. Interestingly, recent evidence points to the bodily fluid-derived exosomes as a potential source of miRNA biomarkers.

**Figure 1:**
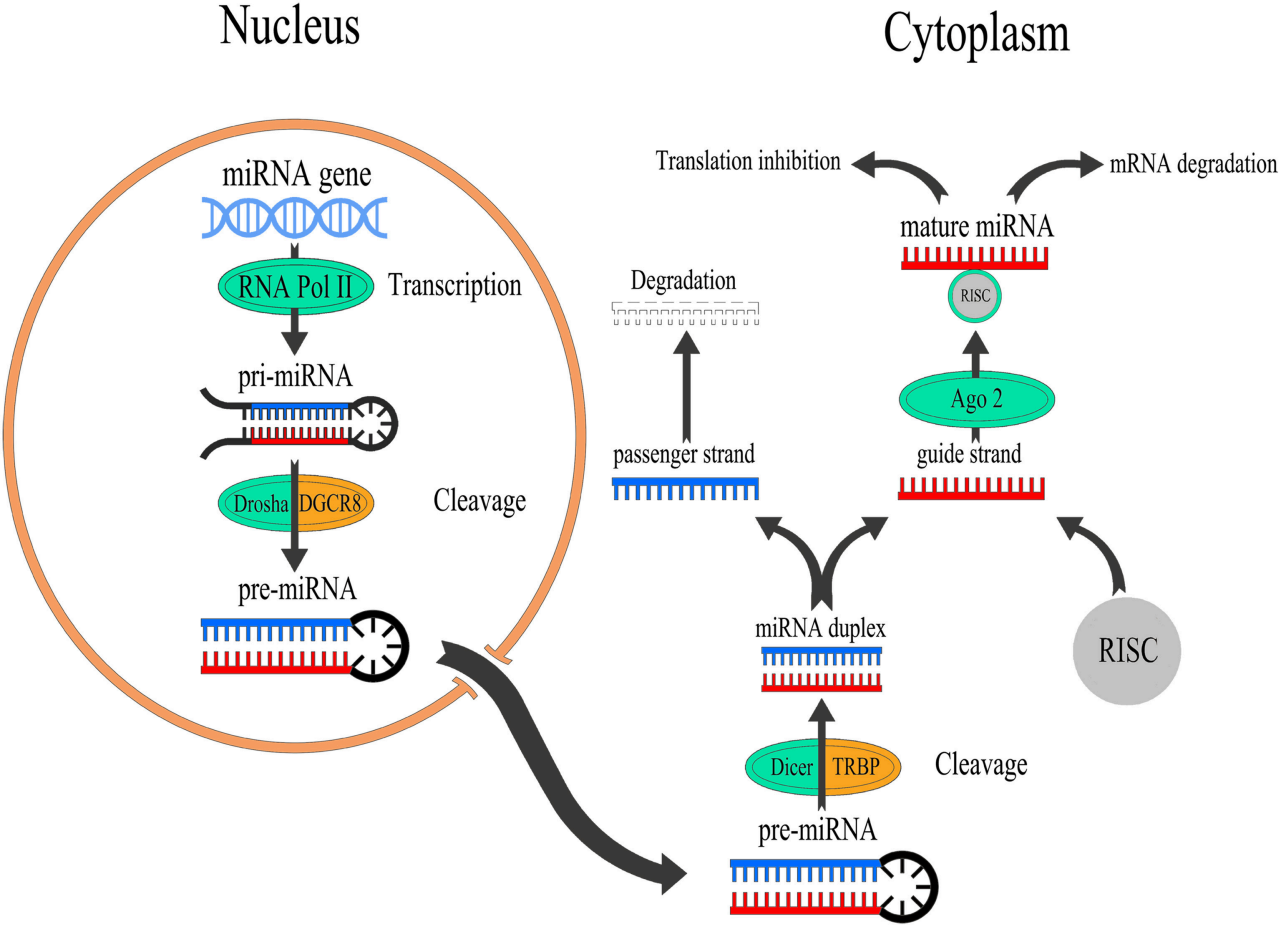
**Overview of miRNA biogenesis and function**. miRNAs are transcribed by RNA Pol-II to form a pri-miRNAs which is cleaved by the nuclease Drosha to produce a pre-miRNA which exports into the cytoplasm to generate mature miRNAs by Dicer. After that, the guide strand of miRNA binds to Ago2 that linked to the RISC to modulate the translation of target mRNAs, while passenger strand of miRNA is typically degraded.

The process of miRNA biogenesis consists of a tightly controlled multiple steps to generate the mature, functional form (Figure 1). Typically, miRNAs are transcribed in the nucleus either form their independent promoters or form introns which lie within genes encoding proteins. Large numbers of miRNAs are transcribed mainly by RNA polymerase II (Pol-II) which generates hairpin structures called primary miRNA (pri-miRNAs) (Lee et al., 2004). Pri-miRNAs are then processed by a series of nuclear enzymes cleavage by the nuclease Drosha microprocessor complex to produce a stem-loop with 60–80 nt called precursor miRNA (pre-miRNA) hairpin (Lee et al., 2003). Subsequently, pre-miRNA is transported into the cytoplasm through exportin-5, importin-β family member, to form the mature miRNA (19–25 nt) by the RNase III enzyme Dicer (Hutvágner et al., 2001). It is worthy of note that lack of Dicer is implicated in various neurological disorders including epilepsy, neuronal and glial dysfunction, and neurodegeneration (Hébert et al., 2010; Tao et al., 2011; McKiernan et al., 2012b). To function, single strand of the mature miRNA, called the guide strand, is bound by a member of Argonaute (Ago) proteins family in order to generate the effector RNA-induced silencing complex (RISC). The other strand of the mature miRNA, called the passenger strand, denoted with a star (miR^*^) is typically degraded. MiRNA can then guide the RISC to their target mRNAs by base-pairing over a minimum 7–8 nt “seed” region of their mRNA targets. Animal miRNAs are usually complementary to a site within the 3′ UTR. However, miRNAs have also been reported to bind to the 5′ end and the open reading frame (Bartel, 2009). This process is followed by supplementary or complementary base pairing that together control target mRNA specificity and affinity. This results in translational repression or degradation thereby decreasing the levels of the target protein ranging from 2- to 10-fold (Fabian et al., 2010). However, miRNAs have also been reported to promote translation under certain cellular conditions (Vasudevan et al., 2007).

## miRNA alterations in epilepsy

Recent works indicated that miRNAs serve pivotal roles in regulating specific genes expression within neurons (Im and Kenny, 2012; Aksoy-Aksel et al., 2014). In epilepsy, miRNAs have been identified as key regulators of protein production during and following seizures. This suggests that miRNAs may affect neuronal excitability and remodeling responses. Nudelman et al. first demonstrated links between altered miRNAs expression and seizures (Nudelman et al., 2010). They studied expression of miR-132 in hippocampus of mice 8 h after pilocarpine-induced SE and found an upregulation in not only level of pri-miR-132 but also in mature miR-132 level. Shortly after, Aronica and colleagues explored miR-146a expression as well as its cellular distribution in both animal model and human TLE (Aronica et al., 2010). The authors found that miR-146a was increased and persistent in reactive astrocytes, suggested that miR-146a is implicated in the controlling of the astroglial inflammatory response occurring in TLE.

### miRNA regulation following SE

The first study about miRNA profiles after SE was by Liu and colleagues. They profiled miRNA expression alterations following KA in rats and detected a significant overlap between the expression of miRNA and mRNA, particularly in gene expression, immunological response, and cell death processes (Liu et al., 2010). The authors found 8 miRNAs were significantly deregulated in both brain tissue and blood samples. Subsequently, multiple studies profiled miRNA responses in the brain using different models, different seizure durations, different time points and different platforms, suggested that miRNAs may play an important role in pathogenesis of epilepsy (Hu et al., 2011, 2012a; Jimenez-Mateos et al., 2011; Song et al., 2011; McKiernan et al., 2012; Pichardo-Casas et al., 2012; Bot et al., 2013; Risbud and Porter, 2013; Gorter et al., 2014; Li et al., 2014; Kretschmann et al., 2015). miRNAs expression Profiles were performed on the lithium-pilocarpine model (Hu et al., 2011, 2012a; Song et al., 2011), systemic pilocarpine (Risbud and Porter, 2013), systemic KA (Liu et al., 2010; McKiernan et al., 2012; Pichardo-Casas et al., 2012), intra-amygdala KA (Jimenez-Mateos et al., 2011), electrical stimulation of angular bundle (Gorter et al., 2014), electrical stimulation of amygdala (Bot et al., 2013; Li et al., 2014), or pilocarpine and self-sustained SE (Kretschmann et al., 2015), with time points ranging from a few hours (McKiernan et al., 2012) to 4 months after induction of SE (Gorter et al., 2014), making direct comparisons difficult. It is worthy of note that a subset of miRNAs is specifically associated with epilepsy include miR-146a, -10b, -34a, -132, -125b, -155, -21, -134, -34c-5p, -212-3p, -219, -218, -204, -184, -98, -381, -181b,c, -221, -222, -196, Let-7i, -9, -23a, -423-3p, -30c, -375, -497, -450a, -296-5p, let-7b, -374, -137, -124, and -199, all of which have been supported by at least two fully independent studies. However, other sets of miRNAs were dysregulated in some studies, but not in others. These variances may be due to varied standards or criteria for the model selection of SE. Furthermore, varied standards to identify significantly dysregulated miRNAs can also result in varied outcomes. Hippocampus is composed of several subfields that have vastly different molecular and functional characterization (Lein, 2004; Greene et al., 2009). Thus, analyzing miRNA expression in tissues obtained from different subfields may lead to different results. Moreover, different species, models, size of samples, brain areas, phases of the disease, array platforms, study design, technical factors, and extraneous effects may also influence the profiling of miRNA abundance.

In 2014, Gorter et al. used Exiqon microRNA arrays to assess the profile of miRNA expression changes in cornu ammonis area 1 (CA1), dentate gyrus (DG) and parahippocampal cortex (PHC), at 1 day, 1 week, and 3–4 months after SE (Gorter et al., 2014). In CA1 and DG, more increased than decreased miRNAs were observed in each phase following SE with highest increased miRNAs in the chronic phase in the DG, while in PHC, most of miRNAs were downregulated. More recently, Kretschmann et al. compared miRNA expression patterns in the whole hippocampus using one acute seizure model evoked by electrical stimulation and two chronic epilepsy models induced either by pilocarpine or self-sustained SE (Kretschmann et al., 2015). Among those screened, an overlap of three miRNAs, miR-30a-5p, miR-142-5p, and miR-331-3p, between the acute and chronic models was reported. Although the three miRNAs were significantly expressed, only miR-142-5p was consistently upregulated in both acute and chronic models. Table 1 provides a summary of the differentially expressed miRNAs in experimental models of epilepsy.

**Table 1:** **miRNA profiling in experimental model**.

**Model**	**Stage (Time point)**	**Platform**	**Aberrantly expressed miRNAs**	**Regulation**	**References**
KA SzPc (mouse)	Acute (8 h)	TaqMan	miR-148b, -376a, -335, -9, -129, -132, -34c, -369-3p, -204, -299-5p, -30a-3p, -7, -34b, -409-5p, -29a, -100, -184, -448, -28, -140, -29c, -375, -31, -130b, let-7f	Up	McKiernan et al., 2012
KA (rat)	Acute (24 h)	TaqMan	miR-298	Up	Liu et al., 2010
			miR-155, -29c, -34b-3p, -98, -122, -203, -450a	Down	
PILO (rat)	Acute (24 h)	Microarray (Agilent)	miR-213, -132, -30c, -26a, -375, -99a, -24, -124a, -22, -34a, -125a, -101-1, -29b, -125b, -199a, -196b, -150, -151, -145	Up	Hu et al., 2011
			miR-29a, -181c, -215, -181b, -25, -10b, -21	Down	
KA SzPc (mouse)	Acute (24 h)	Taqman	miR-10b, -21, -29a, -30e, -125a, -132, -134, -139, -146b, -153, -181c, -199a, -219, -323, -328, -375, -425, -451, -487b, -507, -509, -518d, -532	Up	Jimenez-Mateos et al., 2011
			miR-27a, -101, -103, -107, -127, -133b, -145, -148b, -200a, -326, -330, -374, -381, -422b, -497, -520b, -657	Down	
KA SzPc (mouse)	Acute (24 h)	Taqman	miR-10b, -21, -27a -29a, -30e, -101, -103, -107, -125a, -127, -132,-134, -139, -145, -146b, -148b, -153, -181c, -199a, -200a, -219, -323, -326, -328, -375, -425, -451, -487b, -507, -509, -518d, -532, -629	Up	Jimenez-Mateos et al., 2011
			miR-133b, -330, -374, -381, -422b, -497, -520b, -657	Down	
PILO (mouse)	Acute (24 h)	Microarray (Exiqon)	miR-2137, -21-5p, -711, -212-3p, -882, -1947-5p, -21-3p, -142-5p, -467d-5p, -132-3p, -710, -712-5p, -223-3p, -142-3p, -706, -691, -294-5p, -709, -22-3p, -29a-3p, -431-5p, -126-3p, -29b-1-5p, -483-3p, -29b-3p, -1892, -1957, let-7a-5p, -23a-3p, let-7e-5p, -1935, -19a-3p, -494-3p, -24-2-5p, -335-3p, -146b-5p, -203-3p, -875-3p, -1983, -1897-5p, -17-5p, -146a-5p, -1895, -290-5p, -674-5p	Up	Kretschmann et al., 2015
			miR-331-3p, let-7d-3p, -181c-5p, -324-5p, let-7b-3p, -194-5p, -409-5p, -125b-2-3p, -1941-3p, -467d-3p, -433-3p, -30a-5p, -149-5p, -668-3p, -873-5p, -181d-5p, -467e-5p, -181b-5p, -466d-3p, -761, -1839-3p, -124-5p, -186-5p, -224-5p, -218-2-3p, -881-3p, -330-5p, -491-5p, -337-5p, -380-3p, -542-3p, -361-5p, -181a-1-3p, -669h-3p, -449b, -1224-5p, -374-5p/, -374c-5p, -466c-5p, -208a-3p, -425-3p, -466g, -760-3p, -673-5p, -301b-3p, -742-3p, -488-5p, -216b-5p, -499-5p, -1b-5p, -211-5p, -703, -339-3p, -466l-3p, -302b-5p	Down	
ES (rat)	(7–90 d)	Microarray (Exiqon)	miR-212-3p, -132-3p, -21-5p, -132-5p, -212-5p, -146a-5p, -23a-3p, -370-5p	Up	Bot et al., 2013
			miR-344b-2-3p,-345-5p, -322-5p, -124-5p, -291a-5p, -7a-2-3p, -138-2-3p, -330-3p, -128-3p, -664-3p, -383-5p, -29b-2-5p, -7a-1-3p, -205, -1843-3p, -497-5p, -29c-3p, -7b, -138-1-3p, -505-3p, -30a-3p, -1843-5p, -743a-5p, -186-5p, -103-3p, -324-5p, -324-3p, -124-3p, -330-5p, -582-5p, -107-3p, -146b-5p, -148b-3p, -335, -301a-3p, -29a-5p, -30b-5p, -935, -130a-3p, -190a-5p, -31a-5p, -3580-3p, -29c-5p, -9b-5p, -26a-5p, -218a-5p, -137-3p, -708-5p, -101a-3p, -30a-5p, -33-5p, -30d-5p, -139-5p, -374-5p, -30e-5p, -7a-5p, -551b-3p, -187-3p	Down	
ES (rat)	Latent (7 d)	Microarray (Exiqon)	miR-21-5p^*^, -212-3p, -132-3p, -370-5p^*^	Up	Bot et al., 2013
			miR-7a-2-3p, let-7d-3p, -1843-5p, -1843-5p, -124-3p, -301a-3p, -324-5p, -29a-5p, -708-5p, -935, -92b-3p, -374-5p^*^, -328a-3p, -139-5p, -30d-5p, -33-5p^*^ -187-3p^*^, -551b-3p, -7a-5p	Down	
PILO (mouse)	Chronic (28 d)	Microarray (Exiqon)	miR-135b-5p, -132-3p, -199a-5p, -23a-3p, -129-5p, -129-2-3p, -669c-5p, -467e-5p, -212-3p, -203-3p, -467c-3p, -467e-3p, -455-3p, -466f-3p, -669f-3p, -222-3p, -297c-5p, -27a-3p, -297a-5p, -467g, -22-5p, -494-3p	Up	Kretschmann et al., 2015
			miR-325-3p, -345-5p, -1949, let-7f-1-3p, -191-5p, -350-3p, -331-3p, -875-3p, -138-1-3p, -181a-5p, -34b-3p, -409-5p, -338-5p, -676-3p, -187-3p, -551b-3p, -674-5p, -194-5p, -324-5p, let-7d-3p, -210-3p, -140-3p, -298-5p, -130a-3p, -29b-1-5p, -92b-3p, -330-3p, -431-3p, -767	Down	
ES (rat)	Chronic (30 d)	Microarray (Exiqon)	miR-146a-5p, -132-5p, -21-5p^*^, -212-5p, -23a-3p, -34a-5p, -370-5p^*^, -34b-5p, -24-2-5p, -433-3p, -34c-5p	Up	Bot et al., 2013
			miR-29c-5p, -30a-5p, -374-5p^*^, -33-5p^*^, -218a-5p, -187-3p^*^, -30e-5p, -352, -29b-3p	Down	
PILO (rat)	Chronic (60 d)	Microarray (μParaflo)	miR-99a, -134, -127, -379, -137, -324-5p, -27b, -383, -132, -24, -29a, -139-5p, -9^*^, -23b, -146a, -140^*^, -23a, -126	Up	Song et al., 2011
			miR-98, -352, let-7e, -185, let-7d	Down	
PILO (rat)	Chronic (60 d)	Microarray (Agilent)	miR-146a, -211, -203, -210, -152, -31, -23a, -34a, -27a	Up	Hu et al., 2012a
			miR-138, -301a, -136, -153, -19a, -135b, -325-5p, -380, -190, -542-3p, -33, -144, -542-5p, -543, -296	Down	
ES (rat)	Chronic (60 d)	NGS	miR-455-3p, -345-3p, -423-3p, -54, -365-5p,	Up	Li et al., 2014
			miR-296-5p	Down	
ES (rat)	Chronic (3–4 m)	Microarray (Exiqon)	miR-126, -126^*^, -129, -129-2^*^, -132, -132^*^, -135a, -140, -140^*^, -143, -144, -145, -146a, -152, -199a-3p, -199a-5p, -204, -21, -210, -212, -212^*^, -214, -223, -23a, -23b, -24, -24-1^*^, -24-2^*^, -27a, -27b, -322, -34a, -34b, -34c, -378, -451, -542-3p	Up	Gorter et al., 2014
			miR-139-5p, -187, -218a, -551b, -935	Down	
PILO (rat)	Acute (4 h)	Microarray (Exicon)	miR-132, -184, -214, -516b, -470, -518c^*^, -519e^*^, -519c-5p, -503, -M1-4, -583, -BART8^*^, -637, -M1-8, -K12-6-3p, -S1-5p, -713, -658, -K12-8, -467b^*^, -132^*^, -934, -125b-1^*^, -193a-5p, -21^*^, -185^*^, -183^*^, miRPlus_27560, -423-5p, -711-765, Plus_28431, -675-5p, -498, -883a-5p, -149^*^, Plus_42487, -516a-5p, -miR-881^*^, -212, -885-3p, -483-5p, -642, -485-3p, -625^*^, -30c-2^*^, -371-5p, Plus_42745, -187^*^, -921, Plus_42793, -300^*^, -638, -882, ebv-miR-BHRF1-1, -25^*^, -193b^*^, -300, -763, -294^*^, -518a-5p, -551a, -198, -204^*^	Up	Risbud and Porter, 2013
			ND	Down	
PILO (rat)	Acute (48 h)	Microarray (Exicon)	miR-21, -155, -M1-8, -685, -S1-5p, -_SNORD3@, -881^*^, -483-5p, Plus_42793, -300	Up	Risbud and Porter, 2013
			miR-9, -126, let-7i, -130a, let-7d, -103, -107, -125a-5p -137, -148a -154, -181b, -191, -181a^*^ -218, -22, -221, -222, -26a, -299-5p, -29b -29c, -30a,-376b, -377, -382, -378, -503, -519d, -519e, -98, -99b, -207, -300, -30e^*^, -329, -345-5p, -380-3p, -433^*^, -434-3p, -434-5p, -151-5p, -333, -336, -352, -450a, -7-1^*^, U6-snRNA-1, -138, -301a, -369-5p, -195, -322, -10a, -376a^*^, -539, -487b, -541, -29c^*^, -361-5p, -374b, -381, -664, -124, -411, -7-2^*^, -30b, -629^*^, -691, -92b, -let-7a, -let-7d, -let-7f, -let-7a^*^, miRPlus_17891, -99b^*^, -491-3p, -186, -450a, -let-7c, -let-7i, -100, -148b, -30a^*^, -let-7g, -742, -374a, -190, -139-5p, -363^*^, -744, -891a, -30e, -872, -741, -667, -let-7b, -9^*^, -768-5p, -340, -125b, -804, -384-3p, -342-3p, let-7f, -126^*^, -505, -382^*^, -376c^*^, -598, let-7e, let-7g, -485-5p, -181c, -136^*^, -22^*^, -933, -494, -589, -379^*^, -26b, -384, -634, -503^*^, -376c, -140-3p, -145, let-7c-1^*^, -505, -337-3p, -328, -495, -136^*^, -325, -99a, -887, -33a, -34b, -668, -138, let-7e^*^ -872^*^, -665, -347, -125b^*^, -411^*^, -337-3p, -29b-2^*^, -582-5p, -150,-149, -218-2^*^, -597, -888^*^, -127-3p, -28-5p, -181d, -384-5p, -497, -433, miRPlus_42856, -743b, -181a, -337-5p, -138-1^*^, -331-3p, -875-3p, -379^*^, -467e^*^, -124^*^, -185, -327, -551b, -29a^*^, -30c, -409-5p, -582-3p, -134, -BART17-5p, -101b, -323-3p, -742,	Down	
PILO (rat)	Latent (3 w)	Microarray (Exicon)	miR-21, -142-3p, -155, -19a, -19b, -583, -BART8^*^, -685, -S1-5p, -K12-8, -193a-5p, Plus_17952, _SNORD2, -142-5p, -675-5p, -498, Plus_42487, -516a-5p, -20b, -106a, -20a, -17, -423-3p, -371-5p, -20b, -25^*^, -550, -34a	Up	Risbud and Porter, 2013
			miR-299-5p, -7-1^*^, -615-3p	Down	
PILO (rat)	Acute (2 h)	qPCR	miR-124, -134, -132,-21, -183, -135a, -125b, -27a, -9, -155, 30c	Up	Omran et al., 2012; Ashhab et al., 2013a,b; Peng et al., 2013; Alsharafi and Xiao, 2015
			miR-221, -222, -138, -181a, -128	Down	
	Latent (3 w)		miR-132, -146a, -27a	Up	
			miR-21, -221, -222, 30c	Down	
	Chronic (60 d)		miR-124, -134, -132,-21, -183, -135a, -125b, 27a, -9, -146a, -155, -181a, -30c	Up	
			miR-138, -221,- 222, -128	Down	

*PILO, pilocarpine; KA, kainic acid; SE, status epilepticus; ES, Electrical stimulation; NGS, next-generation sequencing; SzPc, seizure preconditioning; ND, not detected*.

* *means the passenger strand of the mature miRNA*.

In the brain, endogenous programs of neuroprotection can be elicited by exposure to brief, non-harmful seizures (preconditioning), which is considered as a potential means to protect against epilepsy (Jimenez-Mateos and Henshall, 2009). Two previous studies undertook miRNA expression profiling at two different time points in experimental models of epileptic tolerance: after seizure preconditioning but before SE (McKiernan et al., 2012) and after SE in previously preconditioned mice (Jimenez-Mateos et al., 2011). In response to SE, most of expressed miRNAs were upregulated, this was drastically reduced in the tolerance mice, in which only 18% of the expressed miRNAs were upregulated and 82% were downregulated (Jimenez-Mateos et al., 2011). This indicates that genomic responses to SE are reprogrammed by seizure-preconditioning, which was also observed for protein coding genes in preconditioning-induced ischemic tolerance (Stenzel-Poore et al., 2007). It has been found that epileptic tolerance features transcriptional suppression due to increasing miRNA levels by preconditioning seizures that would later decrease mRNAs of protein-coding genes (Jimenez-Mateos et al., 2008). In the cornu ammonis area 1 (CA3) subfield of the mouse hippocampus, seizure preconditioning upregulated levels of 25 mature miRNAs with the greatest upregulation detected for miR-184, whereas no miRNA was significantly down-regulated (McKiernan et al., 2012).

### miRNA regulation in human epilepsy

In the intervening years, six studies have undertaken the miRNA expression profiling either in brain tissue samples (Kan et al., 2012; McKiernan et al., 2012b; Kaalund et al., 2014; Zucchini et al., 2014) or serum (Wang et al., 2015a,b) of patients with epilepsy (Table 2), along with study on individual miRNAs in human epilepsy (Aronica et al., 2010; Jimenez-Mateos et al., 2012; Omran et al., 2012; Ashhab et al., 2013a,b; Peng et al., 2013; Alsharafi and Xiao, 2015). Specifically, miRNAs expression profiles were performed on patients with mesial temporal lobe epilepsy (mTLE) (Kan et al., 2012; Kaalund et al., 2014), temporal lobe epilepsy (TLE) (McKiernan et al., 2012b), or Drug-resistant epilepsy (Zucchini et al., 2014; Wang et al., 2015b). Each study provided a new insight into the potential functions of miRNAs in the pathogenesis of human epilepsy. The first study was conducted by Kan et al. (2012). In their study, human hippocampal tissues either with or without sclerosis were analyzed to profile human miRNA. The authors found that 51 miRNAs were significantly dysregulated. Several of these miRNAs were decreased in neuron and increased in glia, others were highly expressed in the nucleus which suggested a novel function of these miRNAs or a defect in their biogenesis. Furthermore, astrocytes and the immune response can be regulated by differentially expressed miRNAs in mTLE (Kan et al., 2012). Widespread reduction of mature miRNAs in patients with mTLE was reported by McKiernan and co-workers. They indicated that miRNA were collapsed as an extra patho-mechanism in mTLE due to loss of Dicer expression which was reduced in this study (McKiernan et al., 2012b).

**Table 2:** **miRNA profile in human epilepsy**.

**Patients**	**Specimen**	**Platform**	**Aberrantly expressed NAs**	**Regulation**	**References**
mTLE	Hippo	Microarray (Exiqon)	miR-9, let-7f, -16, -17, -20a, -26b, -27a, -92b, -99a, -106a, -126, -129-3p, -135a, -190, -193a-3p, -195, -203, -301a, -340, -362-3p, -374a, -374b, -597, -625, -660, -1297	Up	Kan et al., 2012
			miR-141, -146b-3p, -185,-214, -220c, -490-3p, -635, -637, -642, -665, -920, -934, -1255a, -1304, -1469, -1973, Plus-F1021, Plus-E1026, Plus-E1185, Plus-E1212, Plus-E1232	Down	
TLE	Hippo/TL neocortex	Taqman	miR-133b, -182, -200c, -650, -380-5p, -594, -223, -501, -203, -451	Up	McKiernan et al., 2012b
			miR-181d, -130b-3p, let-7g, -411-5p, -335-5p, -210, -103a-3p, -23b-3p, let-7d, -25-3p, -340, -100-5p, -99a-5p, -27b-3p, -126-5p, -324-5p, -21-5p, -425, -92, -497-5p, -125b-5p, let-7f-5p, -187, -330-3p, -29c, -423, -130a-3p, -433, -95, let-7b-5p, -30d-5p, -106b-5p, -9^*^, -301a-3p, -30b-5p, -432-5p	Down	
DRE	HGC	Microarray (Agilent)	miR-1234, -1281, -30c-1^*^, -92b, -191^*^, -1238	Up	Zucchini et al., 2014
			miR-3607-5p, -219-5p, -338-3p, -590-5p, -487a, -3659	Down	
mTLE	Hippo	Microarray (Exiqon)	miR-302b, -432^*^, -299-3p, -501, -515-3p, -197, -502, -383, -183, -519d, -493, -338-5p, -10b, -184, -141, -375, -200a, -130b, -182, -193b, -361, -368, -519e^*^, -429, -199a,	Up	Kaalund et al., 2014
			miR-140^*^, -204, -211, -490, -218	Down	
Epilepsy	Serum	NGS (Illumina)	miR-144-5p, -15a-5p, -181c-5p, -194-5p, -889-3p, -96	Up	Wang et al., 2015a
			let-7d-5p, -106b-5p, -130a-3p, -146a-5p	Down	
DRE	Serum	NGS (Illumina)	miR-574-5p, -67, novel -9	Up	Wang et al., 2015b
			miR-194-5p, -204-5p, -221-5p, -301a-3p, -30b-5p, -342-5p, -3605-5p, -4446-3p, -598-3p, -874-3p, -889-3p, novel-451	Down	
mTLE	Hippo	qPCR	miR-183, -135a, -125b, -27a, -146a, -155, -9, 181a, 124, 134, -21, -132, -30c,	Up	Omran et al., 2012; Ashhab et al., 2013a,b; Peng et al., 2013; Alsharafi and Xiao, 2015
			miR-128, -138, -221, -222	Down	

*mTLE, mesial temporal lobe epilepsy; DRE, drug resistant epilepsy; Hippo, hippocampus; T.L., temporal lobe; HGC, hippocampal granule cells; NGS, next-generation sequencing*.

* *means the passenger strand of the mature miRNA*.

In a recent study, more than 1000 human miRNAs were profiled in 14 human hippocampi obtained from patients with intractable TLE and hippocampal sclerosis (HS) either with type-2 granule cell pathology in 7 cases or with no granule cell pathology in the other 7 cases. They found that, 6 miRNAs were increased and 6 miRNAs were decreased as well. Of these, miR-487a was validated using qPCR and ANTXR1 was identified as a probable target.

In another study, miRNA expression profiling demonstrated that 30 miRNAs were statistically expressed in biopsy specimens obtained from patients with mTLE/HS (Kaalund et al., 2014). Of these, miR-218 and miR-204 were statistically decreased in mTLE/HS, and both were highly expressed in the different hippocampal subfields. Meanwhile, miR-204 and miR-218 expressions were dynamically altered during hippocampal development in pigs. Taken together, these findings indicate that both miR-204 and miR-218 may play a pivotal role in the molecular mechanisms involved in the progression of pathology in mTLE/HS (Kaalund et al., 2014). Surprisingly, the most hippocampus-enriched miRNAs (let-7 family, miR-9 and miR-124) were not detected in most of epilepsy studies (Eacker et al., 2011; Shinohara et al., 2011). Thus, it requires researchers in the field of epilepsy to re-mine their previous data to identify the underlying mechanisms.

### Comparison of experimental to human epilepsy

There are multiple challenges to overcome in order to successfully compare such outcomes. First, different miRNAs have different expression patterns in different tissues and models (Pichardo-Casas et al., 2012). This means that comparing the miRNA expression pattern between animal models and human epilepsy is unreliable. Second, different miRNAs varies with phase of disease. Avoiding comparison between different phases of epilepsy in animal models is therefore a significant challenge to overcome. Specifically, only deregulated miRNAs in chronic phase of epilepsy in animal models can be compared with the expression levels of those miRNAs deregulated in human epilepsy. Third, different miRNAs varies with ethnicity and age. This means that data obtained from immature animal model and children with epilepsy cannot be compared with those obtained from adults. In addition, different patient race studies cannot be compared as well. Finally, the comparison is also challenging due to the discrepancies among the different criteria (i.e., 1.5- vs. 2.0-fold of changes) used to select the differentially expressed miRNAs in these profiling studies. These outstanding obstacles still make a direct comparison difficult. Therefore, compare data with similar methodology, model, species, tissues, phase, and race may lead to more reliable outcomes. However, no perfect agreements in the literature about miRNAs comparison rules have been provided.

In this section, we attempted only to connect recent available profiling studies to highlight the similarities and differences in the miRNAs found altered in animal models and human with epilepsy.

The miRNA expression levels were considered to be selected only if they met the following criteria: (i) validating in at least 2 profiling studies; (ii) showing no expression variation or discrepancies in all previously reported datasets. According to these criteria, we observed that there is an overlap between these previously reported datasets. We found that 75 miRNAs (43 downregulated and 32 upregulated) fulfilled all the criteria in animal models. In human epilepsy, only 16 miRNAs met our criteria, 6 downregulated and 10 upregulated miRANs as well (Table 3). Of these, downregulation of let-7d-5p and miR-301a-3p levels were corroborated between animal models and human epilepsy studies. Likewise, miR-92b and miR-130a-3p were found to be upregulated in human epilepsy but were downregulated in animal model of epilepsy, while miR-204 was found to be downregulated in human epilepsy and upregulated in animal models. Interestingly, all these 5 miRNAs were found to be deregulated in the chronic phase of epilepsy in animal models, which make our comparison meaningful. Therefore, these 5 miRNAs may play different roles, individually or in combination, in the pathogenesis and treatment of epilepsy. However, further studies on these miRNAs are required to identify the underlying mechanisms.

**Table 3:** **Common differentially expressed miRNAs**.

**Human studies/experimental models**	**Upregulated miRNAs**	**Downregulated miRNAs**
Experimental models	miR-17, -21-5p, -23a/b, -24, -24-2-5p, -27b, -31, -34a/c, -125a, -129, -132, -132^*^, -140, -142-3p/5p, -146a, -148, -152, -184, -199a, **-204**, 212-3p/5p, -214, 233, -296, -375, -451, -455-3p, -711, -882	**Let-7d-5p/**3p, let-7f, -29c-3p/5p, -30a-5p, -30e-5p, -34b-3p, **-92b**, -98, -124-5p, **-130a-3p**, -138, -181a/b/d, -185, -186, -187, -190, -191, -218a, **-301a-3p**, -325, -330-3p/5p, 331-3p, 337-5p, -345-5p, -361-5p, -374-5p, -380-3p, -381, -409, -450, -497, -505, -551b-3p, -582-5p, -664, -742, -875-3p, -935
Human epilepsy	miR-9, -27a, **-92b**, **-130a-3p**, -135a, -141, -182, -183, -203, -501	**let-7d-5p**, -30b-5p, -106b-5p, **-204**, **-301a-3p**, -490

*The Let-7d-5p, miR-92b, miR-130a-3p, miR-204, and miR-301a-3p were expressed in both experimental models and human epilepsy, which are highlighted*.

* *means the passenger strand of the mature miRNA*.

Among different research groups, multiple dramatic discrepancies were observed regarding the expression of miRNAs during epilepsy. Some of these discrepancies were definitely due to variations in the methodology, while most of these discrepancies were a mystery. For example, in animal profiling studies, we found clear discrepancies in the expressions of miR-126, -19a, -99a, -378, -153, and -137 that may arise from the difference in phases of epilepsy. Likewise, discrepancies in the expression of miR-324-5p, -210, let-7e, -153 and -181c could be related with differences in the species being employed, while discrepancies in miR-181c, -153 -155, -542-3p, and -203 expressions might be related with differences in the model being used. On the other hand, there were clear unexplained discrepancies for miR-21, 27a, -29a, -34b, -124, -134, -139-5p, -145, and -99a expressions. Notably, these miRNAs are found to be expressed in most studies.

In human epilepsy, we observed that miR-340 expression was upregulated in the tissues obtained from patients with mTLE in a microarray profiling study (Kan et al., 2012), whereas downregulated expression was reported in tissues obtained from TLE patients in a profiling study using Taqman array (McKiernan et al., 2012b). Therefore, the difference of tissues and array platforms might explain the discrepancies. The expression of miR-146a was upregulated in tissues obtained from children with mTLE using qPCR (Omran et al., 2012), but when next-generation sequencing (NGS) platform was used its expression was downregulated in serum obtained from adult patients with epilepsy (Wang et al., 2015a). For miR-141, no any variations were reported which may explain the discrepancies.

Up to now, problems are recognized in the interpretation of results from expression profiling studies of human brain specimens. First, the majority of studies have employed tissues that were obtained either from autopsies or epileptic tissue is derived from surgery specimens and normal tissue from autopsies. Second, post-mortem modifications and durations have found to vastly change the molecular constituents of the obtained tissue, making the data questionable. Finally, different regions in the brain have different cellular constituents that definitely alter in the course of diseases. Therefore, researchers should reconsider their methodology to overcome these challenges.

## miRNAs as biomarkers of epilepsy

To date, epilepsy diagnosis is based mainly on EEG and neuroimaging, which appear at comparatively late phases in the pathogenesis. These methods are not only expensive but also do not provide high-resolution data sets. This urges the need for noninvasive, easy detected, sensitive and specific biomarkers to improve the current diagnosis and to predict the treatment outcome of epilepsy. Owing to chemistry of miRNAs, stability in biofluids and resistance to nuclease digestion in plasma, serum, CSF, and other bodily fluids (Chen et al., 2012; Boon and Vickers, 2013), they have emerged as potential biomarkers of many neurological disease states, including Parkinson's disease (Shtilbans and Henchcliffe, 2012), multiple sclerosis (Gandhi et al., 2013), Alzheimer's disease (Tan et al., 2014), and epilepsy (Wang et al., 2015a,b). In epilepsy, most of the available target and genome-wide miRNA expression profiling studies were based on animal or human hippocampal tissues. Only few profiling studies have focused on the use of miRNAs as biomarkers. Liu et al., in their brilliant study, showed that three miRNAs (miR-333, -685, and -298) were dysregulated at 24 h after KA-induced SE in whole-blood miRNA expression profile, indicating that these three miRNAs can be considered to be used as diagnostic biomarkers for epilepsy (Liu et al., 2010). In a study by You et al., increased miR-196b were reported as a novel biomarker for the diagnosis and prediction of epilepsy associated with glioma (You et al., 2012). In our previous study, we observed that miR-34a, -22, -125a, and -21 were significantly deregulated in hippocampal tissues as well as in rat peripheral blood at 24 h following SE, representing a role for these miRNAs in the future diagnosis of epilepsy (Hu et al., 2011). Significant alterations of miR-21 expression were also found at latent phase which was coincided with the change observed in other brain areas (Gorter et al., 2014). Gorter et al. examined the expression levels of miR-21, -146a, and -142, in plasma at different phases of TLE (Gorter et al., 2014). The upregulation of miR-146a expression in plasma appeared comparatively later in the chronic phase, while miR-142 was increased during the acute phase. Recently, circulatory miRNA expression profiling have provided valuable molecular markers for detection of epilepsy (Wang et al., 2015a,b). Wang and co-workers used Illumina HiSeq 2000 technology to profile the genome-wide miRNA expression in serum from 30 patients with epilepsy (partial and generalized) (Wang et al., 2015a) and 30 patients with drug-resistant epilepsy (Wang et al., 2015b). In the former study, According to their criteria (fold-change >2.0 or < -2.0; *p* < 0.05; miRNA copies ≥ 10), 6 down-regulated miRNAs and 4 up-regulated miRNAs were reported. Among these 10 dysregulated miRNA, 4 miRNAs (Let-7d-5p, miR-106b-5p, -130a-3p, and -146a-5p) were significantly upregulated, and 2 miRNAs (miR-15a-5p and -194-5p) were significantly decreased in human epilepsy relative to normal controls. Moreover, miR-106b-5p was considered as the best diagnostic biomarker for epilepsy due to its high sensitivity and specificity. In the latter study, 12 miRNAs were found decreased and 3 were found increased in drug-resistant patients relative to drug-responsive group. Following miRNAs confirmation by qRT-PCR, miR-194-5p, miR-301a-3p, miR-30b-5p, miR-342-5p, and miR-4446-3p were statistically dysregulated in drug resistant group when compared to drug-responsive patients and control group. Of these 5 miRNAs, miR-301a-3p was reported as the best diagnostic biomarker for drug-resistant epilepsy with highest sensitivity and specificity. Interestingly, this miRNA was significantly correlated with seizure severity in a direct negative manner. Notably, miR-301a-3p and miR-106b-5p were also reported to be downregulated in hippocampal tissues of patients with TLE (McKiernan et al., 2012b). Therefore, both are not only potential diagnostic biomarkers but also may play a neuroprotective role during TLE.

Interestingly, exosomes represent another important source of miRNA as a biomarker. Various exosomal proteins have been reported as critical biomarkers in a variety of neurological diseases such as Alzheimer disease, Parkinson's disease, prion disease, and glioblastoma (Rajendran et al., 2006; Vella et al., 2007; Skog et al., 2008; Alvarez-Erviti et al., 2011). Hu et al. and co-workers observed that miR-29b can be released in exosomes to control HIV which is associated with attenuated platelet-derived neurotrophic factor (PDGF) in adjacent neurons (Hu et al., 2012b). Therefore, exosomes carried genetic information have potential clinical utility for biomarkers tools.

## Therapeutic aspects of miRNAs

Since alterations in miRNA expression profiles have been observed after SE in experimental epilepsy models (Liu et al., 2010; Hu et al., 2011, 2012a; Jimenez-Mateos et al., 2011; Song et al., 2011; McKiernan et al., 2012; Pichardo-Casas et al., 2012; Bot et al., 2013; Risbud and Porter, 2013; Gorter et al., 2014; Li et al., 2014; Kretschmann et al., 2015) and in patients with TLE (Kan et al., 2012; McKiernan et al., 2012b; Kaalund et al., 2014; Zucchini et al., 2014; Wang et al., 2015a,b), a subset of miRNAs are under investigation as potential regulators of a wide variety pathways involved in epilepsy such as neuroinflammation, blood brain barrier (BBB) dysfunctions, apoptosis, ion channels, tumors, axonal guidance, cell proliferations, neuronal function, and synaptic plasticity (Chen et al., 2007; Friedman et al., 2007; Delaloy et al., 2010; Magill et al., 2010; Smrt et al., 2010; Zhao et al., 2010; Tivnan et al., 2011; Iyer et al., 2012; Sano et al., 2012; Henshall, 2013; Shaltiel et al., 2013; Dombkowski et al., 2014; Jiang et al., 2014; Lopez-Ramirez et al., 2014; Zheng et al., 2014; Haenisch et al., 2015; Kamphuis et al., 2015; Rom et al., 2015; Xiang et al., 2015) (Table 4).

**Table 4:** **miRNA involved in epilepsy and possible targets**.

**miRNA**	**Regulation**	**Targets**	**Related pathways to epilepsy**	**References**
146a	Up	IL-1β	Inflammation	Aronica et al., 2010; Iyer et al., 2012; Omran et al., 2012
221, 222	Down	ICAM1	Inflammation	Kan et al., 2012; Ashhab et al., 2013a
155	Up	TNF-α	Inflammation, BBB	Ashhab et al., 2013b; Lopez-Ramirez et al., 2014; Kamphuis et al., 2015
98	Down	CCL2, CCL5	Inflammation, BBB	Rom et al., 2015
34a	Up	Bcl-2, Caspase-3, *TSC1* 3′ UTR	Apoptosis, Neuronal differentiation, and synaptic signal transmission	Tivnan et al., 2011; Hu et al., 2012a; Sano et al., 2012; Henshall, 2013; Dombkowski et al., 2014
423-3p	Up	Caspase-3, Caspase-6	Apoptosis	Li et al., 2014
296-5p	Down	Caspase-3	Apoptosis	Li et al., 2014
132	Up	CREB, AChE, BDNF/TrkB	Dendritic growth, arborization, cholinergic tone, and calcium channel	Friedman et al., 2007; Fabian et al., 2010; Magill et al., 2010; Shaltiel et al., 2013; Xiang et al., 2015
134	Up	Limk1	Dendritic spine	Jimenez-Mateos et al., 2012
487a	Down	ANTXR1	Granule cell dispersion	Zucchini et al., 2014
219	Down	CaMKIIγ	NMDA receptor	Zheng et al., 2014
218	Down	GRM1, SLC1A2, ROBO1, GNAI2	Axonal guidance and synaptic plasticity	Kaalund et al., 2014
204	Down	GRMI	Axonal guidance and synaptic plasticity	Kaalund et al., 2014
23a	Up	TSC1 3′ UTR	Synaptic signal transmission and remodeling	Song et al., 2011; Dombkowski et al., 2014
21	Up	NT-3	Neurite outgrowth	Risbud and Porter, 2013
Let-7i	Up	Unknown	Neuronal death	Chen et al., 2007
184	Up	AKT2	Apoptosis, Interleukin signaling, and cell proliferation	Krichevsky et al., 2006; McKiernan et al., 2012
9	Up	Unknown	Nerve regeneration, epilepsy Network, cell proliferation, cell migration, and neural differentiation	Krichevsky et al., 2006; Delaloy et al., 2010
196b	Up	PCNA	Cell proliferation	You et al., 2012
let-7b	Up	Nuclear receptor TLX	Cell proliferation	Liu et al., 2010; Zhao et al., 2010
137	Up	Ubiquitin ligase mind bomb-1	Neuronal maturation	Smrt et al., 2010
124	Up	Unknown	Neural differentiation and cell proliferation	Peng et al., 2013
199	Down	HIF-1α	Neuronal cell death and angiogenesis	Jiang et al., 2014
212-3p, 132-3p	Down	SOX11	Neural differentiation and neuronal excitability	Haenisch et al., 2015

Two direct strategies to develop miRNA-based therapeutics were identified: mimics or agomirs to restore a loss of function of miRNAs and increase their effective levels. The other called inhibitors or antagomirs which intended to block endogenous levels of miRNAs to increase expression of its mRNA targets. Several functional studies using agomirs/antagomirs reported miRNAs as novel potential approaches to treat epilepsy. For instance, antagonizing miR-132 significantly decreased the hippocampal damage, indicating that miR-132 overexpression may play a role in neuronal death during SE (Jimenez-Mateos et al., 2011). miR-132 modulates hippocampal mRNAs such as acetylcholinesterase (AChE) or the GTPase activator p250GAP (Hanin and Soreq, 2011; Shaltiel et al., 2013). In addition, miR-132 has been previously linked to synaptic plasticity (Vo et al., 2005; Wayman et al., 2008). In contrast, antagonizing miR-184 enhanced cell injury after preconditioning by KA (McKiernan et al., 2012). This suggests that miR-184 may serve to enhance cell survival post-SE. In another study, antagomirs against miR-134 markedly reduced spine density on pyramidal neurons in CA3 and suppressed seizure severity after intra-amygdala KA injection (Jimenez-Mateos et al., 2012). Moreover, the miR-134 antagomirs prevented KA toxicity *in vitro* in a Limk1-dependent manner. This indicates that targeting of miR-134 may also be a promising direct neuroprotective and not as simple as it might seem.

It is well-established that neuronal injury after seizures results in direct excitotoxic necrosis. For example, miR-34a has been found to directly target p53, suggesting a proapoptotic effect of this miRNA in cells (Hermeking, 2010). In our previous work, we found that antagonizing miR-34a after intra-cerebroventricular injection of pilocarpine in rats attenuated the expression of activated caspase-3 by targeting bcl-2. We suggested that miR-34a may participate in promoting neuronal survival and reduce neuronal death or apoptosis (Hu et al., 2012a). However, Sano et al. did not find any of these effects of miR-34a after intra-amygdala injection of KA in mice (Sano et al., 2012). Other studies also observed that miR-34a participates in regulating neuronal differentiation and synaptic signal transmission (Tivnan et al., 2011; Sano et al., 2012; Henshall, 2013; Dombkowski et al., 2014). The different results may be due to the varied animal models employed and varied time points analyzed. In a recent study, miR-219 was found to be downregulated in the hippocampus at days 1, 3, 7, and 10 after intra-cerebroventricular injection of KA. In the same study, silencing of miR-219 by its antagomir led to seizure behaviors, abnormal cortical EEG recordings. Meanwhile, treatments with the miR-219 agomir suppressed seizures and abnormal EEG recordings. The authors provided evidence that in miR-219 play a pivotal role in ameliorating epilepsy via regulating the CaMKII/NMDA receptor pathway (Zheng et al., 2014). In fact, the mechanism of miRNA hypofunction by antagomirs is based on its molecular chemistry via mediating the degradation and sequestration mechanisms (Stenvang et al., 2012). These results suggest antagomirs/agomir targeting these miRNA may represent novel approach to epilepsy treatment.

Increasing evidence has shown that miRNAs play a universal role in neuro-inflammation which contributes to the development of the epileptogenic process (Vezzani and Granata, 2005; Vezzani et al., 2013). Related researches were aimed to evaluate the role of inflammation-related miRNAs in epilepsy. Omran et al. examined the correlation between the expression of IL-1β and miR-146a in immature rats and children with mTLE. The authors found that both IL-1β and miR-146a are upregulated and variable depending on the disease phase (Omran et al., 2012). In a previous study, it was claimed that miR-146a was linked to astrocyte-mediated inflammatory response (Iyer et al., 2012). Ashhab and colleagues aimed to detect the relationship between TNF-α and proinflammatory miR-155 in immature rats and children with mTLE (Ashhab et al., 2013b). They found that these two markers had similar expression patterns in the three phases of mTLE development, which were upregulated in the seizure-related phases but not in the seizure-free phases. The co-expression of TNF-α and miR-155 in astrocyte indicates a direct correlation during mTLE development (Ashhab et al., 2013b). In 2012, miR-221 and miR-222 were also found to target ICAM1 in astrocyte level in patients with mTLE, suggesting a potential role of these inflammation-related in mTLE (Kan et al., 2012). In 2013, Ashhab and colleagues aimed to explore the dynamic expression of miR-221 and miR-222 in the three phases of TLE and in children with TLE. They demonstrated that both miR-221 and miR-222 were downregulated in the three phases of TLE in animal models and in children with epilepsy (Ashhab et al., 2013a). In the same study, inflammation-related miR-138 was decreased in the seizure-related phase but not in the latent phase, whereas inflammation-related miR-181a was decreased in the acute phase, normal in the latent phase and increased in the chronic phase (Ashhab et al., 2013a). In our previous work, we investigated the dynamic expression pattern of inflammation-related miR-128, miR-30c, and miR-27a in the three phases of TLE as well as in patients with TLE. We found that miR-128 was downregulated in the acute and chronic phase, while in the latent phase no changes were observed. For miR-30c, upregulation expression was found in the acute and chronic phases and downregulation expression was found in the latent phase of TLE, whereas miR-27a was upregulated in the three phases of TLE. In TLE patients, miR-30c and miR-27a were upregulated, whereas miR-128 was downregulated. These studies not only support the role of inflammation in epilepsy but also provide evidence that modulation of these inflammation-related miRNAs may be a new target for AEDs.

Brain-specific and brain-enriched miRNAs represent promising targets for experimental and therapeutic modulation. Brain-specific miR-124, miR-134, and miR-9 were significantly increased in the seizures related phases (acute and chronic phases), but not in the seizure-free phases (latent phase) in immature rats after SE (Ashhab et al., 2013a; Peng et al., 2013). In the same studies, miR-124, miR-134, and miR-9 were also upregulated in children with mTLE that indicate their possible roles in the treatment of epilepsy in the developing brains. MiR-9 was also implicated in nerve regeneration, epilepsy network, cell proliferation, cell migration, and neural differentiation (Krichevsky et al., 2006; Delaloy et al., 2010). However, further studies are required to determine whether targeting this interesting miRNA by its agomir/antagomir colud affect seizure variables. In our previous study, we revealed upregulation of brain-specific miR-183 and miR-135a as well as of brain-enriched miR-125b in the seizure-related phases and TLE patients but not in the seizure-free phases, suggesting that all may provide a potential therapeutic approach for the treatment of TLE (Alsharafi and Xiao, 2015).

Overexpression of miR-196b has been implicated in pre-operative seizures in patients with glioma, and might be a valuable tool to predict seizure prognosis in patients without pre-operative seizures (You et al., 2012). Therefore, targeting this valuable miRNA may be a new therapeutic approach. Let-7b has also been implicated in regulating neural stem cell proliferation and differentiation by inhibiting nuclear receptor TLX signaling (Zhao et al., 2010). Other miRNAs such as miR-137 and miR-199 have also been implicated in epilepsy (Hu et al., 2011; Jimenez-Mateos et al., 2011; Song et al., 2011; Risbud and Porter, 2013) as well as in regulating neuronal functions (Smrt et al., 2010; Jiang et al., 2014). miR-132-3p and 212-3p have been reported to target SOX11 and contribute to regulate neuronal function as well as neuronal excitability (Haenisch et al., 2015). These finding indicate that modulation of above-mentioned miRNAs, individually or in combination, may exert neuroprotective and disease-modifying effects in epilepsy.

Unfortunately, the BBB potentially restricts the entry of beneficial drugs for treatment of CNS disorders. It is estimated that almost 98% of small molecule drugs fail to cross the BBB, whereas large molecule drugs are unable to cross the BBB. Several strategies have previously been investigated for enabling drugs to across the BBB include chemical and biological delivery systems, BBB disruption, molecular Trojan horses, and particulate drug carrier systems. On the other hand, BBB dysfunction was found to be during epilepsy.

Interestingly, there is growing evidence that endogenous miRNAs can modulate the function of BBB. For instance, several miRNAs including miR-15a, -18b, -26a, -27a/b, -30a/b/c/d/e, -31, -106b, -125a-5p, -155, -195, and -487b were found to increase BBB function in patients with multiple sclerosis (Kamphuis et al., 2015). In a recent study by Lopez-Ramirez et al., miR-155 was also found to negatively regulate BBB under neuroinflammatory conditions (Lopez-Ramirez et al., 2014). More recently, Rom et al. reported that miR-98 and let-7g^*^ contribute to protect the BBB during neuroinflammatory disorders (Rom et al., 2015). Interestingly, several reports have proved that miR-98 is downregulated in the acute and chronic phases of TLE in rats (Liu et al., 2010; Song et al., 2011; Risbud and Porter, 2013). Since neuroinflammation have been found to play a critical role in the pathogenesis of human epilepsy (Vezzani and Granata, 2005; Vezzani et al., 2013), these BBB-associated miRNA may also contribute to protect the BBB function during epilepsy. Therefore, modulating of these miRNAs may be a promising strategy to overcome BBB challenges.

## Concluding remarks

It is evident that miRNAs dysregulation can occur as a result of epilepsy. The previous profiling studies provide new insights into the molecular mechanisms associated with the disease progression. miRNA are involved in neuroprotection, CNS development, dendritic spines, neuronal differentiation, and synaptic signal transmission. Moreover, miRNAs may play special roles in regulating a set of genes associated with a wide range of pathways in the pathogenesis of epilepsy. Thus, a deeper understanding of these biological processes can lead to novel diagnostic and therapeutic strategies. Preclinical studies have shown that pathways such as the Bcl-2, IL-1β, NT-3 and the MAPK pathway that are also involved in miRNA dysregulation may also be promising new horizons for diagnosis and treatment of experimental as well as clinical epilepsy. However, further validations are actively needed.

### Conflict of interest statement

The authors declare that the research was conducted in the absence of any commercial or financial relationships that could be construed as a potential conflict of interest.
